# Anti-Stress Effects of *Tremella fuciformis* Berk. Enzymatic Extracts: A Preclinical Study

**DOI:** 10.3390/nu17050914

**Published:** 2025-03-06

**Authors:** Gahye Moon, Nodir Rustamov, Junhang Park, Hanseul Park, Kumju Park, Eun Hye Choi, Yoon-Seok Roh

**Affiliations:** 1 College of Pharmacy, Chungbuk National University, Cheongju 28160, Republic of Korea; moongahye12@gmail.com (G.M.); nodirrustamov.nr@gmail.com (N.R.);; 2 Pulmuone Institute of Technology, Cheongju 28220, Republic of Korea; kjparka@pulmuone.com

**Keywords:** chronic stress, neuroprotection, corticosterone (CORT), *Tremella fuciformis*

## Abstract

**Background/Objectives:** Chronic stress disrupts neurochemical balance, triggers inflammation, and compromises neuronal integrity, contributing to the development of stress-related disorders. This study aimed to evaluate the preventative effects of *Tremella fuciformis* Berk (TF) enzymatic extracts on chronic restraint stress (CRS)-induced behavioral, neurochemical, and inflammatory dysfunctions in mice. **Methods:** Male C57BL/6N mice were administered TF at doses of 50 mg/kg and 100 mg/kg daily via oral gavage for 21 days during CRS exposure. Behavioral assessments, including anxiety and depression-like behavior tests, were conducted. Neurochemical and inflammatory markers were analyzed using PCR and ELISA, while histological examinations of hippocampal regions were performed to assess neuronal integrity. In vitro assays evaluated neuronal cell viability, protection against corticosterone (CORT)-induced cytotoxicity, and inhibition of monoamine oxidase (MAO) activity. **Results:** TF supplementation alleviated CRS-induced weight loss, normalized serum CORT levels, increased locomotor activity, reduced immobility time, and decreased anxiety-like behaviors. TF upregulated brain-derived neurotrophic factor (BDNF) mRNA, downregulated pro-inflammatory markers (CXCL2, iNOS, IFNG), and mitigated neuronal apoptosis in the hippocampus. In vitro, TF improved neuronal cell viability, protected against CORT-induced cytotoxicity, and significantly inhibited MAO activity, particularly MAO-A. **Conclusions:** These findings demonstrate the neuroprotective and anti-stress effects of *Tremella fuciformis* Berk enzymatic extracts, supporting its potential as a natural therapeutic intervention for stress-related disorders.

## 1. Introduction

Stress has become increasingly pervasive in modern society, profoundly affecting both mental and physical health [1]. The fast-paced, high-pressure environment of contemporary life contributes significantly to the prevalence of chronic stress, a prolonged and persistent state of tension that disrupts nearly all bodily systems [2,3,4]. Left unmanaged, chronic stress can suppress the immune system, disrupt digestive and reproductive functions, increase the risk of heart attacks and strokes, accelerate aging, and heighten susceptibility to mental health disorders such as depression and anxiety [2,5,6]. These conditions not only impair quality of life but also underscore the importance of understanding the mechanisms through which stress impacts the body and developing effective interventions to mitigate its harmful effects. In the search for effective interventions to combat stress and its harmful consequences, natural products have recently garnered scientific interest for their potential therapeutic benefits [7]. Among these, TF enzymatic extracts, derived from the *T. fuciformis fungus*—commonly known as white fungus or snow mushroom—stand out due to their long history of use in traditional medicine [8,9,10,11]. Renowned for its immune-modulating properties, skin hydration benefits, and potential cognitive enhancement effects, *T. fuciformis* represents a promising candidate for addressing stress-related health challenges [10,12,13]; many of these benefits are linked to its polysaccharides, which have been studied for their antioxidant, anti-inflammatory, and neuroprotective properties—further underscoring its potential in stress management [14,15,16].

TF shows potential as a natural remedy for stress-related disorders by modulating the hypothalamic–pituitary–adrenal (HPA) axis and reducing neuroinflammation [13,17]. Its bioactive components, uridine and mannose, contribute through distinct mechanisms. Uridine regulates glucocorticoid receptor signaling, helping to stabilize CORT levels and prevent excessive stress responses, which can otherwise impair cognition and neuronal function [18,19,20]. It also supports neuronal membrane synthesis and synaptic function, essential for cognitive resilience. Mannose complements these effects by reducing proinflammatory cytokine production, mitigating neuroinflammation, and enhancing BDNF signaling to promote synaptic plasticity and neuronal survival [21,22]. Since chronic stress disrupts HPA axis regulation [23], leading to prolonged CORT elevation and neuroinflammatory responses, TF’s combined effects help restore homeostasis, supporting both neuroprotection and stress resilience. However, scientific evidence supporting its anti-stress benefits remains limited, highlighting the need for further investigation. This study evaluated the anti-stress effects of TF using behavioral tests and complementary biochemical analyses. The chosen behavioral tests were the open field test (OFT), the elevated plus maze (EPM), and the tail suspension test (TST), which together provide a comprehensive assessment of anxiety, depression, and overall activity under chronic stress conditions [24]. The OFT evaluates locomotor activity and anxiety-like behavior, the EPM measures anxiolytic effects through the balance between anxiety-driven avoidance and exploratory behavior, and the TST detects depression-like behavior by measuring immobility time [25,26,27,28]. These tests are valued for their simplicity, efficiency, and versatility, being easy to set up, requiring minimal training, and allowing rapid data collection that is suitable for high-throughput studies. Relying on ethologically relevant behaviors, such as avoidance of open spaces in the EPM, exploration in the OFT, and energy-conserving immobility in the TST, they ensure innate responses that do not depend heavily on training [24,29,30,31]. Their widespread validation across species makes them standard tools for studying anxiety and depression-like behaviors, enabling cross-study comparisons. Additionally, these tests are minimally invasive compared to models using physical harm or extreme stressors, and their clear, quantifiable outcomes make them ideal for statistical analysis and reproducibility. By combining behavioral and molecular approaches, this study provides a holistic evaluation of TF’s anti-stress effects, aiming to validate its potential as an anti-stress agent while contributing to comprehensive strategies for stress management that ultimately seek to improve the well-being of individuals experiencing chronic stress.

## 2. Materials and Methods

### 2.1. Preparation of T. fuciformis Enzymatic Extracts

The enzymatic extract of TF was provided by Pulmuone Co., Ltd. (Seoul, Republic of Korea). Briefly, the dried TF fruiting bodies, cultivated in Fujian Province, China, were milled and extracted with water containing 10% citric acid at 121 °C for 20 min. The hot water extract of TF was adjusted with potassium carbonate to a pH 4.5–5.0 and then hydrolyzed by 10% (*w*/*v*) cellulase MP (Kyowa Hakko Kogyo, Nakano, Japan) at 55 °C. After overnight incubation, the TF extract was filtered, concentrated, and powdered using a spray-dryer with dextrin. The final TF extract was standardized by high-performance liquid chromatography (HPLC) with uridine and mannose, at a concentration of 0.56~0.84 mg/g and 98.4~147.6 mg/g, respectively (Supplementary Material).

### 2.2. Animals and Experimental Design

Male C57BL/6N mice (7 weeks, Samtako Bio Osan, Republic of Korea; total n = 40) were housed under controlled conditions with a 12 h light/dark cycle, a temperature of 23 ± 3 °C, and a relative humidity of 50 ± 5%. After one week without any intervention, the 40 mice were randomly divided into five groups, with 8 mice allocated to each group: (1) control group (control, n = 8); (2) chronic restraint stress group (vehicle, n = 8); (3) low-dose TF group (TF50, n = 8); (4) high-dose TF group (TF100, n = 8); and (5) L-theanine treatment group (n = 8). Each group consisted of different individuals, and there was no overlap of mice between groups. Mice in the control and model groups underwent daily gavage with 0.9% saline (10 mL/kg, JW Pharmaceutical), whereas mice in the TF50, TF100, and L-theanine groups were administered different doses of TF (50 mg/kg, 100 mg/kg) or L-theanine (4.0 mg/kg) daily [13,32,33,34,35]. Drugs were administered from the first day of CRS until the end of the study, and the body weight of each mouse was measured daily. This study was approved by Chungbuk National University IACUC (CBNUR-2234-24, 15 April 2024).

### 2.3. Serum and Hippocampus Collection

The animals were sacrificed under anesthesia (Zoletil-50, Virbac Korea, Seoul, Republic of Korea; 0.1 mL/100 g for rats; 0.05–0.06 mL/10 g of 5X-diluted Zoletil-50 for mice, intramuscular injection). Blood samples were collected immediately via cardiac puncture following anesthesia, and tissues were harvested after all behavioral tests were completed. The collected blood was allowed to clot at room temperature and then centrifuged at 3000 rpm for 10 min to separate the serum. Serum samples were stored at −80 °C until analysis. Hippocampal tissues were carefully dissected and stored at −80 °C for subsequent biochemical and molecular analyses.

### 2.4. Chronic Restraint Stress

The study lasted 21 consecutive days. Each day, mice underwent a 6 h restraint period in 50 mL tubes with holes for ventilation, designed to fit them comfortably, allowing minimal movement and preventing escape. Mice were restrained in tubes for 6 h daily at consistent times and subsequently returned to their home cages. Control mice were maintained under identical housing conditions without restraint exposure [36,37].

### 2.5. Open Field Test

The open field test (OFT) apparatus comprised a circular arena with a diameter of 50 cm and a height of 40 cm. The arena was divided into central and outer zones. The test was conducted under standard laboratory conditions with a 12 h light/dark cycle, a temperature of 22 ± 2 °C, and 60% humidity. Each mouse was placed in the center of the open field arena at the beginning of the test. The behavior of the mice was recorded for 5 min using a video tracking system (SMART-LD program; Panab, Barcelona, Spain) [17]. The measured and analyzed parameters included the total distance traveled, time spent in the central zone, number of entries into the central zone, and the average speed. To ensure consistent conditions and minimize disturbances, the arena was cleaned with 70% ethanol and allowed to dry between trials to remove any olfactory cues left by the previous mouse. The results were used to assess general locomotor activity and anxiety-like behaviors in mice. Mice were divided into four groups: control (0.9% saline), vehicle (0.9% saline), low-dose treatment (50 mg/kg), and high-dose treatment (100 mg/kg). All treatments were administered 30 min before the OFT [31].

### 2.6. Elevated Pulse Maze Test

The elevated pulse maze (EPM) test apparatus comprised a common central platform (5 × 5 cm) extending into two open arms (30 × 5 cm) and two closed arms (30 × 5 × 20 cm) to form a cross. The apparatus was elevated 50 cm above the floor. The test was initiated by placing the mouse on the central platform of the maze, facing an open arm. The following measurements were obtained from the test: time spent in the open and closed arms, number of entries into the open and closed arms, and the percentage of open-arm preferences based on time and entries. The time spent in the open and closed arms and the number of entries into these arms were recorded over a 5 min period using video images (SMART-LD program; Panlab, Barcelona, Spain). Anxiolytic effects were indicated by an increase in the percentage preference for the open arms based on both time and the number of entries, as well as an increase in the time spent in the open arms or the number of entries into the open arms. Overall motor activity was measured as the total number of entries into each arm. All treatments, as described previously, were administered 30 min before the EPM [30].

### 2.7. Tail Suspension Test

The tail suspension test (TST) was performed to assess depression-like behavior in mice by measuring their immobility time, which reflects behavioral despair. In this test, each mouse was suspended by its tail using an adhesive tape positioned approximately 50 cm above the ground. The test lasted for 6 min, during which the behavior of the mice was recorded using video tracking software (SMART-LD program; Panlab, Barcelona, Spain). The key parameter measured was the duration of immobility when the mouse ceased to struggle and remained motionless, which is indicative of a depression-like state. The reduction in the immobility time following treatment was interpreted as an antidepressant-like effect. The TST was conducted under consistent environmental conditions, including controlled lighting and minimal noise, 30 min after administration of the test compounds [28].

### 2.8. Quantitative Real-Time PCR Analysis

Total RNA was extracted from the hippocampus using RiboEX (GeneAll Biotechnology; Seoul, Republic of Korea) and purified using a Hybrid-R kit (GeneAll Biotechnology). The extracted RNA was reverse-transcribed into cDNA using a High-Capacity cDNA Reverse Transcription Kit (Thermo Fisher Scientific, Waltham, MA, USA) and used for quantitative real-time PCR on a CFX Connect Real-Time PCR Detection System (Bio-Rad, Hercules, CA, USA). Target gene expression was normalized to that of glyceraldehyde 3-phosphate dehydrogenase (GAPDH), used as an internal control. The specific mouse primer sequences are GAPDH F: GCCAAGCCTGCTTCTTACTC, GAPDH R: TGAGGGCAATTCCAGCCTTA, BDNF F: CGAGACCAAGTGTAATCCCA, R: TCTATCCTTATGAACCGCCA, iNOS F: ACATCGACCCGTCCACAGTAT, iNOS R: CAGAGGGGTAGGCTTGTCTC, CXCL2 F: CCCCAAGTGTGAAGAACAAGA, CXCL2 R: TGAGTGAAACTGGTTGGGTA, IFNG F: GAACTGGCAAAAGGATGGTGAC, and IFNG R: TTCAAGACTTCAAAGAGTCTGAGG.

### 2.9. CORT ELISA

Serum CORT levels were measured using a commercially available enzyme-linked immunosorbent assay (ELISA) kit (Enzo Life Sciences, CORT ELISA Kit, ADI-900-097) in accordance with the manufacturer’s instructions, with minor modifications to optimize the sample type and volume. Blood samples were collected, centrifuged at high speed at a low temperature to separate serum, and stored at a very low temperature until analysis.

### 2.10. Hematoxylin and Eosin Staining

The samples were mounted on glass slides and stained with H&E 9 (Leica Microsystems, Wetzlar, Germany). Sections were deparaffinized in xylene, rehydrated through graded ethanol, and stained with hematoxylin for 5 min. After rinsing under tap water and differentiating in 1% acid alcohol, slides were blued in ammonia water, counterstained with eosin for 2 min, dehydrated in graded ethanol, and cleared with xylene. Coverslipped slides were observed under a light microscope (Leica DMi1, Leica Microsystems, Germany), and images were captured using a connected digital camera.

### 2.11. MTT Assay

The viability of cells was assessed using the 3-(4,5-dimethylthiazol-2-yl)-2,5-diphenyltetrazolium bromide (MTT) assay with the EZ-Cytox kit (DoGenBio, EZ-Cytox, Cat No. EZ-500), in accordance with the manufacturer’s instructions, with minor modifications to optimize the method for specific cell types and experimental conditions. Primary mouse neurons were used as models in this study. The cells were cultured in a 96-well plate at a density of 1 × 10 cells per well in 100 μL of the appropriate growth medium, at 37 °C in a 5% CO_2_ atmosphere until they reached 70–80% confluency. The viability of the treated cells was calculated as a percentage of that of control cells. Results are expressed as the mean ± standard deviation of triplicate wells.

### 2.12. MAO Assay

Monoamine oxidase (MAO) activity was assessed in primary neurons. Cells were cultured in appropriate growth media under 37 °C and 5% CO_2_ until they reached 70–80% confluency. Subsequently, the cells were homogenized in 0.1 M phosphate buffer (pH 7.4) and centrifuged at 20,000× *g* for 15 min at 4 °C to collect the supernatant. MAO activity was measured using the EZ-Cytox assay (Cat. EZ-500) according to the manufacturer’s instructions, with slight modifications tailored to specific experimental conditions. The experiment was conducted by comparing the following groups: control, CORT (1 μM) treatment, *T. fuciformis* (TF) 1 μg/mL, and 10 μg/mL treatment groups. For each group, the reaction mixture containing the fluorescent substrate kynuramine was incubated at 37 °C for 30 min. The reaction was stopped by adding 2 M NaOH, and the fluorescence intensity was measured using a microplate reader (excitation: 315 nm, emission: 380 nm).

### 2.13. Statistical Analysis

All data are presented as the mean ± standard error of the mean (SEM). Statistical analyses were performed using Student’s t-test and one-way ANOVA, as well as descriptive statistics where appropriate, using GraphPad Prism 9 (GraphPad Software, San Diego, CA, USA). For one-way ANOVA, F-values and degrees of freedom are reported in the corresponding results section. Statistical significance is indicated as * *p* < 0.05, ** *p* < 0.01, *** *p* < 0.001, and **** *p* < 0.0001.

## 3. Results

### 3.1. Effect of TF on Body Weight in Chronically Stressed Mice

**Figure 1:**
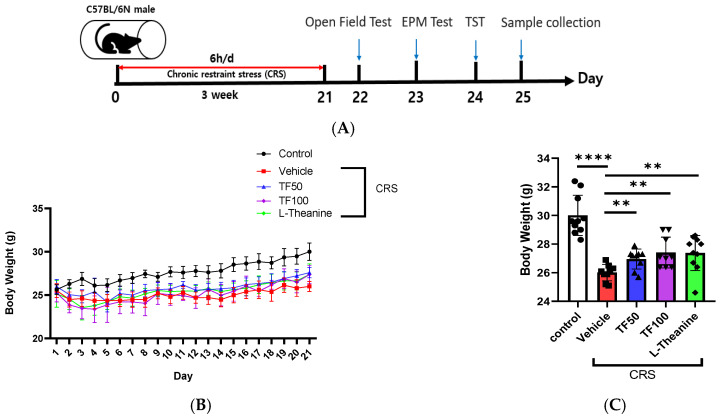
Effect of TF on body weight in chronically stressed mice. (**A**) Schematic representation of the animal experimental model used in this study. (**B**) The body weight of each of the mice was recorded daily during CRS model induction. (**C**) On day 21, the TF50 (50 mg/kg), TF100 (100 mg/kg), and L-Theanine groups all exhibited a significant increase in body weight compared to the vehicle group. Data are presented as means ± SEM (n = 8). Statistical significance is indicated with ** *p* < 0.01, **** *p* < 0.0001.

The TF extract contains bioactive components, including uridine and mannose, which were identified and quantified using the HPLC method (Figure S1) [38,39]. To evaluate this potential, the body weight of each mouse was recorded daily throughout a 21-day chronic restraint stress (CRS) induction period [40]. CRS significantly reduced body weight in the vehicle group compared to the control group, reflecting the physiological burden of chronic stress (Figure 1A). However, treatment with TF at both low (50 mg/kg) and high (100 mg/kg) doses mitigated this weight loss, showing a notable recovery over time (*p* < 0.0001, F = 14.71, F(4, 35) = 1.197). The TF100 group exhibited the most pronounced improvement, with weight trends comparable to those in the L-theanine group, which also displayed weight recovery compared to the vehicle group (*p* < 0.0001, F(20, 861) = 29.91). The effects of treatment varied across time points (*p* = 0.0181, F(80, 861) = 1.383), indicating differences in response depending on CRS duration (Figure 1B).

Further observations revealed that body weight changes were influenced by both treatment and time (*p* < 0.0001, F(4, 861) = 207.3). Mice receiving TF50, TF100, or L-theanine consistently demonstrated greater weight recovery compared to the vehicle group, with the most substantial effects observed in the later stages of the study (Figure 1C). These findings suggest that TF supplementation may help mitigate the adverse physiological effects of chronic stress, supporting its potential as a natural intervention.

### 3.2. TF Reduces Anxiety and Depression-like Behaviors in Mice

The behavioral effects of TF50 and TF100 were evaluated in mice subjected to 21 days of chronic restraint stress (CRS) using three well-established behavioral tests: the open field test, the elevated plus maze, and the tail suspension test, which assess locomotor activity, anxiety-like behavior, and depression-like behavior, respectively [28,41].

**Figure 2:**
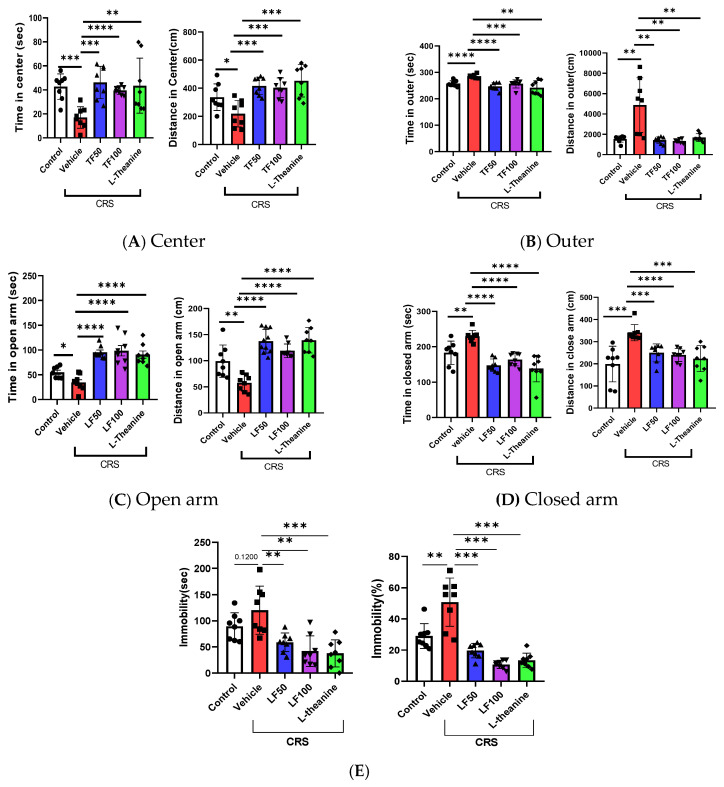
Behavioral tests following treatment with *TF* enzymatic extracts. (**A**,**B**) The OFT was performed after 21 days of CRS treatment, recording the total distance traveled and the time spent in each section during the 5 min test for each mouse. (**C**,**D**) The EPM test was conducted after 21 days of CRS treatment, and the total distance traveled as well as the time spent in each section during the 5 min test were recorded for each mouse. (**E**) The TST was performed after 21 days of CRS treatment, and the immobility time during the 5 min test was recorded for each mouse. Data are presented as means ± SEM (n = 8). Statistical significance is indicated as * *p* < 0.05, ** *p* < 0.01, *** *p* < 0.001, **** *p* < 0.0001.

In the OFT, the total distance traveled, and the time spent in each section of the arena during the 5 min test were recorded. Mice treated with TF50 and TF100 showed a significant increase in total distance traveled compared to the vehicle group, indicating enhanced locomotor activity (F = 8.612, F(4, 35) = 1.388, *p* < 0.0001). Anxiety-like behavior was assessed by analyzing the time spent in the central and peripheral sections of the arena. Mice treated with TF demonstrated altered patterns in time spent in these sections, suggesting a potential anxiolytic effect. Specifically, TF treatment resulted in significantly increased time spent in the center of the arena (F = 6.179, F(4, 35) = 2.789, *p* = 0.0007) and reduced time in the outer sections (F = 8.901, F(4, 35) = 5.517, *p* < 0.0001) (Figure 2A,B). These changes indicate reduced anxiety-like behavior.

In the EPM test, TF50- and TF100-treated mice exhibited a significant increase in total distance traveled (F = 19.93, F(4, 35) = 1.765, *p* < 0.0001), consistent with the OFT results. Additionally, TF-treated mice spent significantly more time in the open arms than in the closed arms (time in open arms: F = 17.82, F(4, 35) = 1.800, *p* < 0.0001; time in closed arms: F = 15.35, F(4, 35) = 1.067, *p* < 0.0001), further indicating reduced anxiety-like behavior (Figure 2C,D).

Similarly, in the TST, which measures depression-like behavior, immobility time was significantly reduced in TF-treated mice compared to the vehicle group (F = 10.45, F(4, 35) = 2.789, *p* < 0.0001), with corresponding reductions in immobility (F = 29.53, F(4, 35) = 3.033, *p* < 0.0001) (Figure 2E). These findings collectively demonstrate that TF alleviates CRS-induced anxiety- and depression-like behaviors while enhancing locomotor activity, highlighting its potential as an intervention for stress-related behavioral impairments.

### 3.3. TF Prevents CRS-Induced Neurochemical and Inflammatory Dysregulation

Chronic stress disrupts the regulation of CORT and inflammatory responses. Prolonged stress elevates CORT levels through HPA axis activation, contributing to inflammation and disrupting homeostasis [42]. In this study, CORT levels were measured using an ELISA, revealing significant increases under stress conditions. TF administration effectively mitigated these increases in a dose-dependent manner, with high-dose TF demonstrating a significant reduction in CORT levels (F = 6.752, F(4, 34) = 6.814, *p* = 0.0004). These findings indicate that TF can restore hormonal balance disrupted by stress.

**Figure 3:**
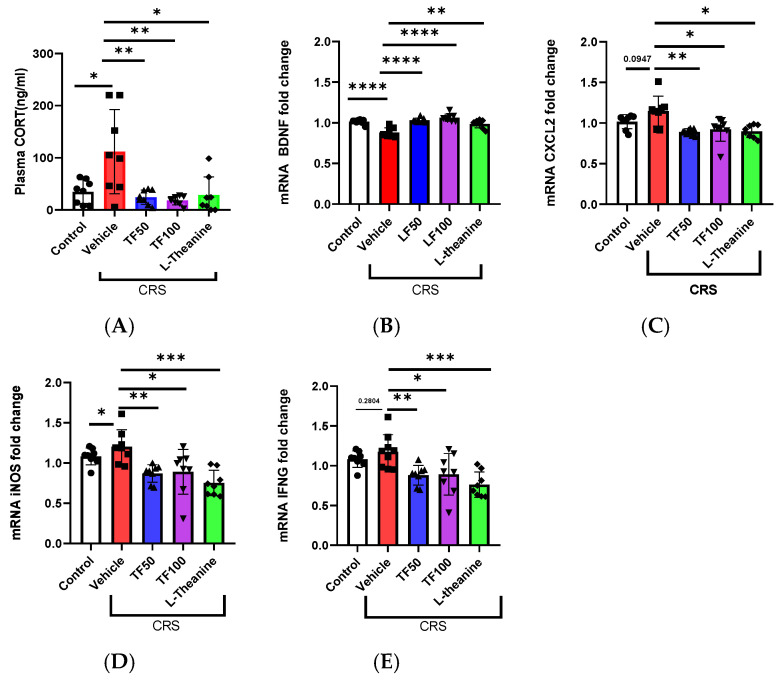
Effects of *TF* on stress-induced neurochemical and inflammatory markers. (**A**) CORT levels were significantly increased under stress and were dose-dependently reduced by TF administration. (**B**) Stress decreased BDNF mRNA expression, but TF administration restored its levels, suggesting neuroprotective effects. (**C**–**E**) TF treatment reduced the expression of inflammation markers, including CXCL2, iNOS, and IFNG, indicating anti-inflammatory effects. Data are presented as the means ± SEM (n = 8). Statistical significance is indicated with * *p* < 0.05, ** *p* < 0.01, *** *p* < 0.001, **** *p* < 0.0001. mRNA expression levels were normalized to mouse GAPDH levels.

Additionally, BDNF mRNA expression, a critical marker of neuronal health and plasticity, was significantly decreased under stress but was restored following TF administration (F = 20.5, F(4, 34) = 0.951, *p* < 0.0001), suggesting its role in promoting neuroprotection and recovery from stress-induced neuronal damage (Figure 3C).

Furthermore, TF exhibited anti-inflammatory effects by reducing stress-induced expression of inflammation markers, including CXCL2 (F = 6.654, F(4, 34) = 1.014, *p* = 0.0004), iNOS (F = 7.665, F(4, 34) = 1.042, *p* = 0.0002), and IFNG (F = 7.017, F(4, 34) = 1.382, *p* = 0.0003). These results indicate TF’s potential to alleviate chronic stress-related inflammation and improve overall health (Figure 3D,E).

### 3.4. Histopathological Analysis of Hippocampal Regions Following TF Treatment

**Figure 4:**
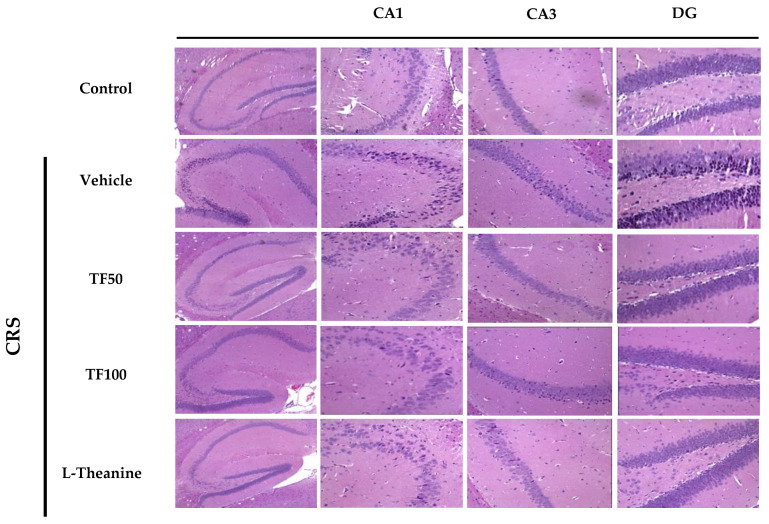
Neuroprotective effects of TF in hippocampus under chronic restraint stress. H&E staining of hippocampal regions (CA1, CA3, and dentate gyrus) in vehicle and TF-treated groups under chronic restraint stress (CRS) shows nuclear condensation in vehicle group neurons, indicating stress-induced apoptosis. TF-treated groups exhibit reduced nuclear condensation, suggesting neuroprotective effects.

Chronic restraint stress (CRS) significantly affects the hippocampus, particularly the CA1, CA3, and dentate gyrus (DG) regions, which are critical for stress response and neurogenesis. The CA1 region, highly sensitive to stress, often shows neuronal damage, while the CA3 region and DG exhibit stress-induced neuronal loss and structural disruptions. H&E staining revealed nuclear condensation, a marker of neuronal apoptosis, in these regions under CRS (Figure 4).

In contrast, TF treatment effectively mitigated these detrimental effects. In TF-treated groups, the extent of nuclear condensation in the CA1, CA3, and DG regions was significantly reduced, suggesting that TF preserves neuronal integrity and exerts neuroprotective effects against stress-induced apoptosis (Figure 4). These findings underscore TF’s potential to counteract CRS-induced neuronal damage, protecting key hippocampal regions involved in memory and stress regulation.

### 3.5. Neuroprotective Effects of TF on CORT-Induced Stress in Neuronal Cells

To expand upon the study, we investigated whether TF exhibits neuroprotective properties similar to L-theanine, a compound known for its protective effects against CORT-induced toxicity [43]. Previous research has demonstrated that L-theanine improves neuronal cell viability under CORT-induced stress conditions and inhibits monoamine oxidase (MAO-A and MAO-B) activity, which are associated with stress-related neurodegenerative processes [43,44,45].

**Figure 5:**
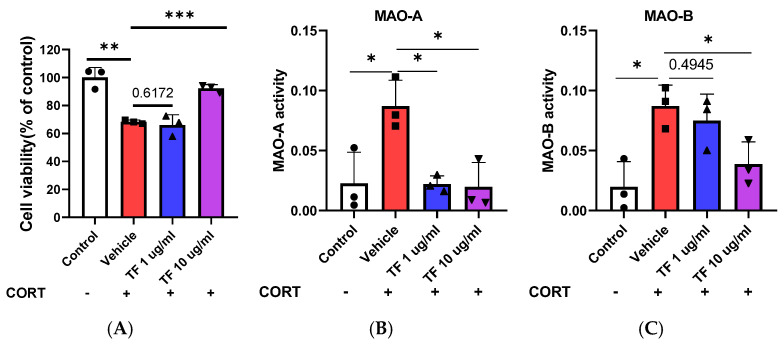
Effects of TF enzymatic extracts on primary neuron cell viability and MAO activity. (**A**) TF increased cell viability in primary neurons treated with 1 μg/mL and 10 μg/mL compared to the CORT 1 μM-treated vehicle group. (**B**) TF significantly reduced MAO-A and MAO-B (**C**) activity, with the greatest effects observed at a concentration of 10 μg/mL, emphasizing its neuroprotective potential. Data are presented as the means ± SEM (n = 3). Statistical significance is indicated with * *p* < 0.05, ** *p* < 0.01, *** *p* < 0.001.

To determine whether TF could replicate these effects, we assessed primary neuronal cell viability and MAO activity under four treatment conditions: control, CORT (1 μM)-treated vehicle, TF at 1 μg/mL, and TF at 10 μg/mL. TF demonstrated significant improvements in cell viability (F = 29.72, F(3, 8) = 0.5617, *p* = 0.0001) (Figure 5A) and reductions in MAO activity, particularly at the 10 μg/mL dose. Specifically, MAO-A activity was significantly reduced (F = 8.141, F(3, 8) = 0.2569, *p* = 0.0082), as was MAO-B activity (F = 7.488, F(3, 8) = 0.01859, *p* = 0.0104) (Figure 5B,C). These findings suggest that TF, like L-theanine, effectively mitigates CORT-induced neurotoxicity, potentially through the inhibition of MAO-A and MAO-B activity, reinforcing its potential as a neuroprotective agent in stress-induced neuronal damage.

## 4. Discussion

This study highlights the potential of *Tremella fuciformis* as a preventative treatment for alleviating the negative effects of chronic stress by protecting the brain and preventing depression-like symptoms. TF demonstrates its effectiveness by counteracting stress-induced behavioral, physiological, and neurochemical disruptions. Chronic restraint stress (CRS) significantly reduced body weight in untreated mice, a hallmark of stress-induced physiological imbalance [46]. TF administration effectively mitigated this weight loss, showing comparable results across all doses and achieving similar effectiveness to the positive control, L-theanine. These findings suggest that TF can help maintain systemic health under chronic stress conditions [47]. Beyond its systemic benefits, TF exhibits robust protective effects on brain function and behavioral assessments revealed that TF-treated mice experienced reduced anxiety- and depression-like symptoms, as evidenced by increased locomotor activity and greater time spent in central and open areas in the open field test and elevated plus maze, respectively. Similarly, immobility time in the tail suspension test—a marker of depression-like behavior—was significantly lower in TF-treated groups. These behavioral improvements are supported by TF’s ability to restore neurochemical balance, particularly by normalizing elevated CORT levels caused by CRS. Furthermore, TF upregulated BDNF mRNA expression, a key marker of neuronal health and plasticity, underscoring its neuroprotective potential [48]. TF’s anti-inflammatory effects were evident in the reduction in CRS-induced inflammatory markers, including CXCL2, iNOS, and IFNG, which are linked to systemic and neuroinflammation [49]. Collectively, these findings indicate that TF not only prevents CRS-induced behavioral impairments but also mitigates neuroinflammation and preserves neurochemical homeostasis.

Histopathological and cellular analyses further confirm TF’s neuroprotective properties. CRS-induced neuronal damage, marked by nuclear condensation in hippocampal regions, was significantly reduced in TF-treated groups. Preservation of neuronal integrity in the CA1, CA3, and dentate gyrus regions highlights TF’s ability to counteract apoptosis and structural damage critical for memory and stress regulation [50]. In vitro studies provided additional evidence, showing that TF enhanced cell viability, reduced cytotoxicity in primary neurons in a dose-dependent manner, and inhibited monoamine oxidase (MAO) activity, particularly MAO-A, which is closely associated with stress-induced neurodegeneration [51,52]. By modulating neurochemical and enzymatic pathways, TF demonstrates a strong potential to counteract neuronal damage, prevent depression-like behaviors, and support overall brain health under chronic stress conditions.

This study highlights TF’s robust efficacy in mitigating the physiological, behavioral, and neurochemical effects of chronic stress. However, several areas warrant further investigation. While using a whole TF extract aligns with traditional medicinal practices, identifying its specific bioactive components [15,53], such as uridine and mannose, could enhance precision in TF-based interventions. Additionally, the molecular mechanisms underlying its neuroprotective and anti-stress properties remain unclear, necessitating further research into relevant cell signaling pathways.

Extrapolating these findings to humans requires careful consideration. This study was conducted exclusively in male mice, limiting generalizability [54]. Future research should examine sex-specific differences by including female subjects and addressing interspecies variations in stress physiology, metabolism, and TF bioavailability [16]. Clinical trials are crucial to confirm TF’s safety and efficacy in humans, considering factors such as age, sex, and health status [54,55]. These considerations highlight the importance of further research to validate TF’s potential as a preventative intervention for stress-related disorders. Despite these gaps, the study demonstrates TF’s significant potential as a natural remedy for stress-related disorders, paving the way for further development and clinical investigation.

## 5. Conclusions

This study demonstrates the potential of *Tremella fuciformis* Berk. enzymatic extracts as an effective natural intervention for managing stress-related disorders. *Tremella fuciformis* alleviates chronic restraint stress-induced anxiety, depression, weight loss, and CORT dysregulation, while also improving neuroprotection by upregulating brain-derived neurotrophic factor expression and preserving hippocampal integrity. Furthermore, it exhibits anti-inflammatory properties by reducing markers such as CXCL2, iNOS, and IFNG, and enhances neuronal health by improving cell viability and inhibiting monoamine oxidase, particularly monoamine oxidase-A. These multifaceted effects highlight its capacity to address the diverse impacts of chronic stress on the body and mind. With its demonstrated ability to modulate stress-related pathways and improve neuronal health, TF holds great promise for incorporation into holistic strategies aimed at enhancing resilience against chronic stress and improving overall well-being.

## Data Availability

The original contributions presented in this study are included in the article/Supplementary Materials. Further inquiries can be directed to the corresponding author(s).

## Supplementary Materials

The following supporting information can be downloaded at: https://www.mdpi.com/article/10.3390/nu17050914/s1.
