# Supplementary material for: Anti-Stress Effects of Tremella fuciformis Berk. Enzymatic Extracts: A Preclinical Study
Source: Nutrients. 2025 Mar 6;17(5):914. doi: 10.3390/nu17050914 (PMC11901780; doi:10.3390/nu17050914)
Supplement: Supplementary file 1 [file nutrients-17-00914-s001.zip › nutrients-3487484-supplementary.pdf]

Supplementary material

Figure S1.

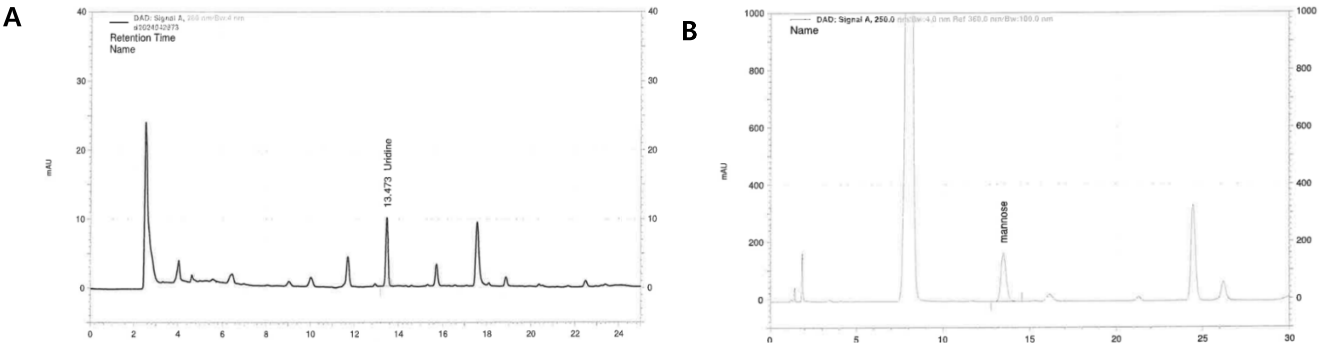

Figure S1. High performance liquid chromatograms of the TF extract (A) for uridine and (B) for mannose. Analytical conditions are described below. Uridine was analyzed using a Agilent HPLC system with a diode array detector (Agilent technologies, Santa Clara, CA, USA) and Capcell pak C18 MG III column (250 × 4.6 mm, 5 μm; Shiseido, Tokyo, Japan). The gradient for the analysis was set at 0-6 min, 100:0(A:B); 20 min, 80:20; 22-25 min, 100:0, using water as solvent A and methanol as solvent B, at a flow rate of 1 mL/min. The detection wavelength was set at 260 nm. Mannose was analyzed using a Agilent HPLC system with a diode array detector and Eclipse XDB C18 column (150 × 4.6 mm, 5 μm; Agilent). The gradient was set at 0 min, 98:2(A:B); 15 min, 95:5; 20~25 min, 83:17; 30 min, 98:2, using 85% of potassium dihydrogen phosphate(0.05M, pH 6.9) in acetonitrile as solvent A and 60% of potassium dihydrogen phosphate(0.05M, pH 6.9) in acetonitrile as solvent B, at a flow rate of 1 mL/min. The detection wavelength was set at 250 nm.

Table S1. Descriptive Statistics Table.

Group	Mean Body Weight (g)	Std. Deviation	Std. Error of mean	Minimum (g)	Maximum (g)	Range (g)
**Control**	30.15	1.557	0.5503	28.3	32.4	4.1
**Vehicle**	26.03	0.6159	0.2177	25.1	26.9	1.8
**TF50**	26.93	0.7421	0.2624	25.7	27.9	2.2
**TF100**	27.45	1.144	0.4044	26.4	29	2.6
**L-Theanine**	27.50	1.333	0.4713	24.6	28.6	4

The table summarizes the descriptive statistics for body weight measurements (in grams) across different experimental groups: Control (no stress), Vehicle (CRS without treatment), TF50 (50 mg/kg treatment), TF100 (100 mg/kg treatment), and L-Theanine (positive control). Key metrics include the mean body weight, variability (standard deviation and range), and precision of the mean (standard error). The data illustrates the effects of CRS and the recovery potential of TF and L-Theanine treatments.

Table S2. General composition of *Tremella fuciformis* extract.

Extract Content	Content amount
Carbohydrate(%)	78.02%
Crude protein (%)	7.42%
Crude Fat (%)	0.35%
Moisture (%)	6.5%
Ash content (%)	7.71%
Sodium (mg/100g)	228.22mg/100g

The table summarizes the nutritional composition of *Tremella fuciformis* extract value, macronutrient distribution (carbohydrates, protein, fat), moisture content, ash content, and sodium concentration per 100 grams of extract.
